# A Frailty-Based Plasma Proteomic Signature Capturing Overall Health and Well-Being in Older Adults

**DOI:** 10.1093/geroni/igaf122.2086

**Published:** 2025-12-31

**Authors:** Fangyu Liu, Sanish Sathyan, Toshiko Tanaka, Nir Barzilai, Sofiya Milman, Keenan Walker, Joe Verghese

**Affiliations:** National Institute on Aging, United States; Stony Brook University, Stony Brook, New York, United States; National Institute on Aging, Baltimore, Maryland, United States; Institute for Aging Research, Bronx, New York, United States; Albert Einstein College of Medicine, Albert Einstein College of Medicine, New York, United States; National Institute on Aging, Baltimore, Maryland, United States; Stony Brook University, Stony Brook, New York, United States

## Abstract

Frailty is an age-related syndrome characterized by an increased vulnerability to adverse health outcomes in the face of stressors. By deriving a blood-based proteomic signature for frailty, the current study aimed to enhance the understanding of frailty biology and derive a person-specific predictor for risk of frailty and other adverse age-related health outcomes. Penalized regression method was used to derive a 25-proteins signature (proteomic frailty index [pFI]) predictive of the cumulative frailty index (FI) in a training set of participants from the LonGenity cohort (N = 440). The pFI was validated with measured FI at baseline (r = 0.58) in the validation set (N = 440) from the same cohort and in two other independent cohorts: the Atherosclerosis Risk in Communities (ARIC) study (N = 5195, r = 0.61, p < 0.001) and the Baltimore Longitudinal Study of Aging (BLSA, N = 654, r = 0.45, p < 0.001). In all three cohorts, the pFI showed significant associations with age-related conditions including hypertension and diabetes, clinical measures of cholesterols, glucose and lipids, and functional measures of gait speed and grip strength (p < 0.001), after adjusting for demographic factors. In ARIC, the pFI were also significantly associated with cognitive scores (p < 0.001) and incident dementia (HR [95% CI] = 1.07 [1.05-1.09], follow-up: 7.22 [1.82] years). The pFI were also associated with mortality (LonGenity: HR [95% CI] = 1.12 [1.07-1.18]; ARIC: HR [95% CI] = 1.13 [1.12–1.14]; BLSA: HR [95% CI] = 1.11 [1.05–1.17]). In conclusion, we derived and validated a 25-protein signature of frailty that also captures overall well-being, health, and risk for key age-related diseases.

